# Genome-wide transcriptome profiling provides insights into floral bud development of summer-flowering *Camellia azalea*

**DOI:** 10.1038/srep09729

**Published:** 2015-05-15

**Authors:** Zhengqi Fan, Jiyuan Li, Xinlei Li, Bin Wu, Jiangying Wang, Zhongchi Liu, Hengfu Yin

**Affiliations:** 1 Research Institute of Subtropical Forestry, Chinese Academy of Forestry, Fuyang. 311400, Zhejiang, China; 2 Key Laboratory of Forest genetics and breeding, Zhejiang Province. 311400, China; 3 Department of Cell Biology and Molecular Genetics, University of Maryland, College Park, Maryland, USA

## Abstract

The transition from vegetative to reproductive growth in woody perennials involves pathways controlling flowering timing, bud dormancy and outgrowth in responses to seasonal cues. However little is known about the mechanism governing the adaptation of signaling pathways to environmental conditions in trees. *Camellia azalea* is a rare species in this genus flowering during summer, which provides a unique resource for floral timing breeding. Here we reported a comprehensive transcriptomics study to capture the global gene profiles during floral bud development in *C. azalea*. We examined the genome-wide gene expression between three developmental stages including floral bud initiation, floral organ differentiation and bud outgrowth, and identified nine co-expression clusters with distinctive patterns. Further, we identified the differential expressed genes (DEGs) during development and characterized the functional properties of DEGs by Gene Ontology analysis. We showed that transition from floral bud initiation to floral organ differentiation required changes of genes in flowering timing regulation, while transition to floral bud outgrowth was regulated by various pathways such as cold and light signaling, phytohormone pathways and plant metabolisms. Further analyses of dormancy associated MADS-box genes revealed that *SVP*- and *AGL24*- like genes displayed distinct expression patterns suggesting divergent roles during floral bud development.

The decision of when to flower in higher plants is one of the most critical developmental events controlled by multiple genetic factors^1^. The synchronization of environmental conditions and flowering time is critical for the survival of plants, and of great importance in agriculture. The seasonal changes in day length and temperature cause alterations in gene expression that impacts plant growth and development^1^,^2^.

Extensive studies in the annual model species *Arabidopsis thaliana* have built a genetic and molecular framework integrating several signaling pathways to determine the timing of the transition from vegetative to reproductive growth^3^. There are four major flowering pathways including gibberellin pathway, autonomous pathway, vernalization pathway and photoperiod pathway, which have been identified by genetic studies in *A. thaliana*^4^. All of these pathways are integrated by a small number of genes (integrators) to activate floral meristem identity genes which are required for floral organ development^4^. The process of floral organ identity determination has been successfully demonstrated by the well-known ABC model (extended to ABCDE model)^5^,^6^,^7^, and most of A-, B-, C-, D-, E- genes are members of MADS-box domain gene family^5^,^8^. Although there is a great diversity of plant adaptations to seasonal cues, the model systems have provided foundations to uncover the molecular mechanisms of annuals as well as perennials. Some key integrators, such as *CONSTANS* (*CO*) and *FLOWERING LOCUS T* (*FT*), have been shown to possess conserved functions in the control of flowering time in both annuals and perennials^9^.

Different from annuals, flowering control in woody species generally involves floral bud initiation, dormancy, and bud development^2^. Recent advances in plant genome analysis have greatly facilitated our understanding on flowering time regulation in various species, and particularly about the seasonal control of bud dormancy in trees^10^. For instance, the recent whole-genome duplication in poplar has resulted two paralogs of *FT*, *FT1* and *FT2*, and functional analyses revealed that *FT1* expression was induced by cold temperature (during winter) to initiate reproductive buds, while *FT2* was induced by warm temperature and long days (spring/summer) and then promoted vegetative growth^11^. In peach, characterization of the *evergrowing* (*evg*) mutant has revealed multiple MADS-box genes [*Dormancy-associated MADS-box* (*DAM*)] which were required for growth cessation^12^. These DAM genes were found to be members of SVP/AGL24 sub-clade of MADS-box gene family which has been shown to be involved in flowering time control in *A. thaliana*^13^. *SHORT VEGETATIVE PHASE* (*SVP*) and *AGAMOUS-LIKE24* (*AGL24*) were closely related MADS-box genes, but had different roles in regulating flowering time in *A. thaliana*^14^,^15^. Further studies in peach have shown that photoperiod and chilling temperatures were related to the regulation of expression patterns of DAM genes^16^. Overall, substantial progress has been made in understanding the regulation of floral bud development in several model systems such as Arabidopsis and poplar^1^,^3^,^17^. However, our knowledge regarding the mechanisms of environmental control of bud development in trees is less clear. In recent years, the advent of new sequencing technique and *de novo* assembly has presented an unprecedented opportunity for genome-wide studies in non-model species. Indeed, transcriptomics analyses in several tree species have presented a top-down view of gene expression profiles during floral bud development^18^,^19^,^20^. And identification of Differential Expression Genes (DEGs) between various environmental or developmental conditions has emerged as a powerful approach to comprehend the complexity of gene regulatory networks in non-model species. Other systems biology investigations, such as gene co-expression network, Gene Ontology (GO) analysis and gene family analysis, have greatly improved our knowledge of gene functions by interrogating high-throughput transcriptome data.

Genus *Camellia* belonging to Theaceae family contains about 250 species including several important economic plants such as *Camellia japonica* (ornamental), *Camellia sinensis* (beverage), and *Camellia oleifera* (edible oil)^21^,^22^. *Camellia azalea* (also known as *C. changii*) was a newly discovered species of *Camellia* genus which was very unique in flowering time^22^,^23^. Unlike to other species in *Camellia* genus, *C. azalea* blooms during mid-summer while most of the *Camellia* species flower in winter or spring^24^. Due to the stunning habit of *C. azalea* in summer-flowering, it was wildly distributed and introduced into the breeding systems of *Camellia* for generating new and more summer-flowering varieties. Although many attempts of artificial crossing between commercial camellias have been successful in generating hybrids, the desired cultivar with good floral morphology and summer-flowering trait has not yet been produced. Currently, little is known about the molecular mechanism of flowering time regulation in *C. azalea* and how it is different from other relatives in response to environmental factors. In this study, we generated a comprehensive floral transcriptome in *C. azalea* by adopting the second-generation sequencing approach (Illumina Hiseq platform) and *de novo* transcriptome assembly. In total we have generated over 120 million (120 M) high-quality reads and assembled more than 79 thousands unigenes. We characterized the gene expression profiles during three stages of floral bud development by gene co-expression clustering analysis. Furthermore by systematic integrating DEGs and GO enrichment analyses, we found that transition from stage 1 to stage 2 was critical and enriched in flowering timing control; transition from stage 2 to stage 3 involved convergence of multiple signaling pathways (i. e. cold signaling, light signaling, hormone signaling). Finally detailed analysis of MADS-box gene family in *C. azalea* revealed that SVP- and Agl24- like genes were differentially expressed during floral bud development. Our work has presented a comprehensive genomics resource for understanding flowering control in *C. azalea* and provided insights for genetic engineering approach to passing the bottleneck of tradition breeding in ornamental camellias.

## Results

### Identification of morphological characteristics during floral bud development and the de novo assembly of transcriptome in C. azalea

**Figure 1:**
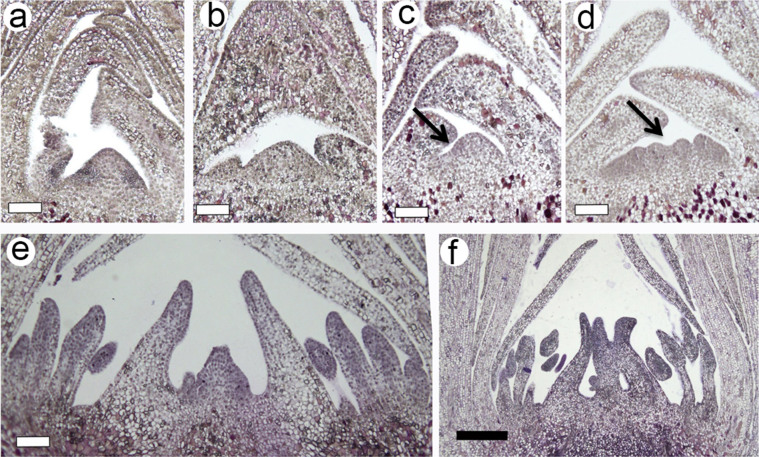
Morphological analysis of floral bud development in *C. azalea*. (a–b), apical buds at stage 1 (less than 4 mm) with no detectable changes to vegetative bud; (c–d), apical buds at stage 2 (5–7 mm) in which reproductive development was initiated. Arrows indicated fasciation of apical meristem suggesting the differentiation of floral organs. (e–f), apical bud at stage 3 in which floral buds began organ differentiation and outgrowth. White bar 50 μm; Black bar 200 μm.

To obtain genomics resources of *C. azalea* during floral development, we set out to generate a comprehensive transcriptome by next-generation sequencing platform. First, we examined the developmental process of floral buds by morphological analysis. The progression of bud development was captured by changes of apical meristem with relevance to bud size (Fig. 1). We collected bud samples from April to mid-June and examined the morphologies through histological sectioning analysis. We showed the development of floral buds and floral organs was associated with bud size (Fig. 1), and we identified three representative stages of floral bud development, including floral bud initiation, floral organ differentiation, and floral bud outgrowth stages (Fig. 1). The apical meristems at first stage (floral bud initiation) were undistinguishable to vegetative meristems (Fig. 1a, b) with bud size less than 4 mm; the second stage (floral organ differentiation) was recognized by fasciation of apical meristems and initiation of floral organs (Fig. 1c, d) with bud size of 5–7 mm; the third stage (floral bud outgrowth) was characterized by the enlargement of floral organ primodia (Fig. 1e, f) with bud size of 8–11 mm.

**Figure 2:**
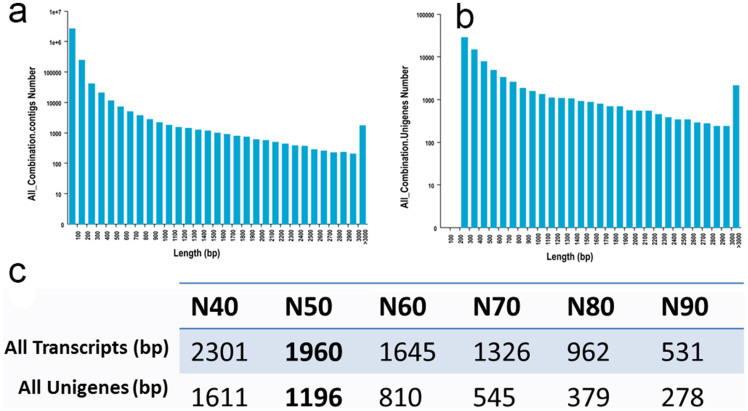
Statistics of *de novo* assembly of transcriptome. (a), Distribution of length of all contigs. (b), Distribution of length of all Unigenes. (c), The statistics of *de novo* assembly of transcriptome.

The mixed samples with all stages of buds were sequenced for generating sequencing reads of *de novo* assembly, and more than 39 M high-quality pair-end reads were retrieved after trimming. The *de novo* assembly was performed by using Trinity software according to standard parameters. The assembly resulted in a total of 213316 transcripts (no less than 200 bp) with a N50 of 1960 bp (Fig. 2a–c), and the unigene dataset included 79363 sequences with a N50 of 1196 bp (Fig. 2c). The scatter plot of size distribution of transcripts and unigenes were shown (Fig. 2a, b). The unigenes were used to preform annotation analysis. The six public databases including COG, GO, KEGG, Swissprot and NR were searched for identifying homologous sequences. In total, 32999 unigenes were found in at least one of these databases (Table 1), and detailed annotation information was listed (Supplementary table S1). To characterize the functional classifications of annotated unigenes, GO and KEGG analyses were performed to access the distributions of functional categories in KEGG and GO databases (Fig. S1).

### Global analysis of transcriptome and clustering of gene expression profiles across three developmental stages reveal distinct co-expression gene clusters

**Figure 3:**
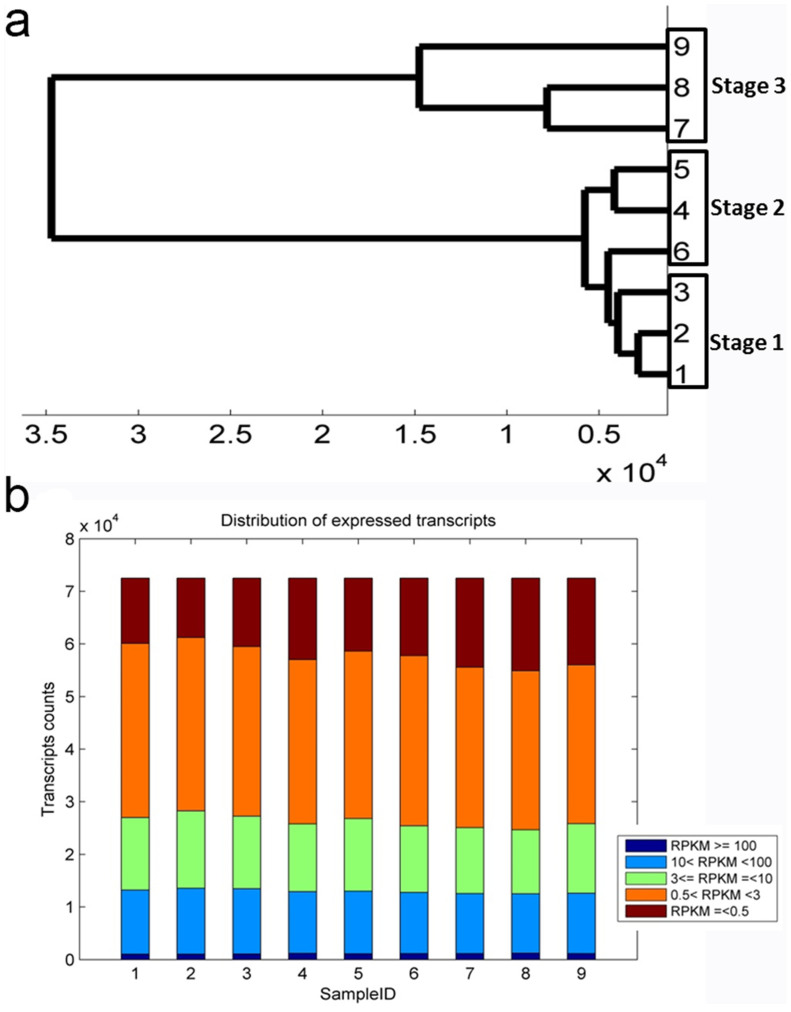
Global analysis of transcriptome datasets of biological replicates and samples. (a), A dengrogram graph showing global relationships of samples. (b), A bar plot describing number of expressed transcripts after filtering. Samples from 1 to 9 stands for S1-rep1, S1-rep2, S1-rep3, S2-rep1, S2-rep2, S2-rep3, S3-rep1, S3-rep2, S3-rep3.

To characterize the developmental events of gene expression profiles, the three stages of floral buds containing three biological replicates for each stage, were sequenced, and generated more than 84 M high-quality reads for quantification of unigenes (Supplementary table S2). The reads were mapped to unigenes and quantified to give the expression abundance of gene expression by Reads Per Kilobase per Million (RPKM)^25^. The RPKM expression data were tested by correlation analysis to evaluate sampling between biological replicates, and all correlation coefficiencies between biological pairs were over 0.93 (Fig. S2). A dendrogram graph was also plotted to examine the relationships between samples, and all biological replicates were clustered together (Fig. 3a). Overall, we found expressions between all biological replicates were highly correlated, and samples of stage 1 and stage 2 were also correlated well (Fig. S2). These data suggested we could estimate quantitatively expression alterations by statistical analysis of this dataset. To depict the global abundance of gene expression, we filtered 6847 transcripts with low expression (sum RPKM value of 9 samples was less than three), and the RPKM values of remainder 72516 transcripts were accessed by box plotting (Fig. 3b).

**Figure 4:**
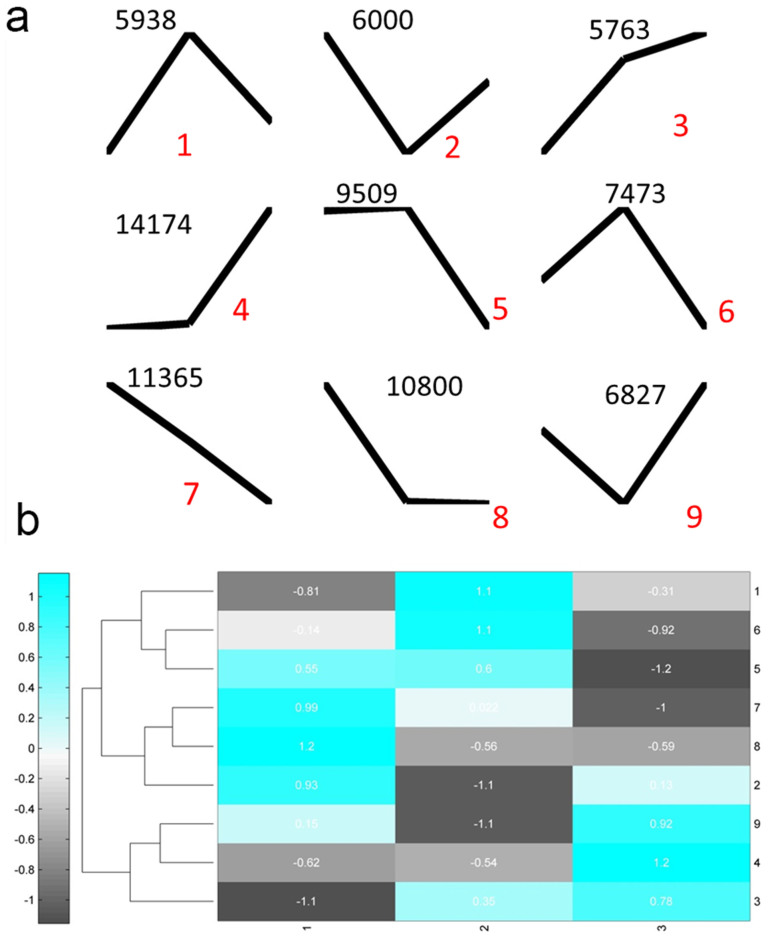
K-Means clustering of gene expression profiles. (a), the centroids of nine clusters with different expression patterns. Numbers on the top are the number of genes for each cluster; and red numbers are cluster labels. (b), a heat map plot of nine clusters display the relative expression levels of centroids. Numbers on the right are corresponding to cluster labels in (a).

To further investigate the gene expression profiles, we performed K-Means clustering of gene expression levels. We identified 9 gene clusters with distinctive expression patterns (Fig. 4a). Cluster 3 and 7 containing 5763 and 11365 transcripts respectively were showing opposite patterns (Fig. 4a). While cluster 3 showed gradual increase from stage 1 to stage 3, cluster 7 showed gradual decrease, indicating different roles regarding the floral bud development. To compare the relationships between clusters, the centroid values which specify the expression patterns of each cluster were clustered, and three major groups were identified (Fig. 4b). And these three groups were characterized by peak expression at three developmental stages (Fig. 4b), indicating that the developmental stages were associated with specific gene clusters.

### Identification of DEGs at different developmental stages

**Figure 5:**
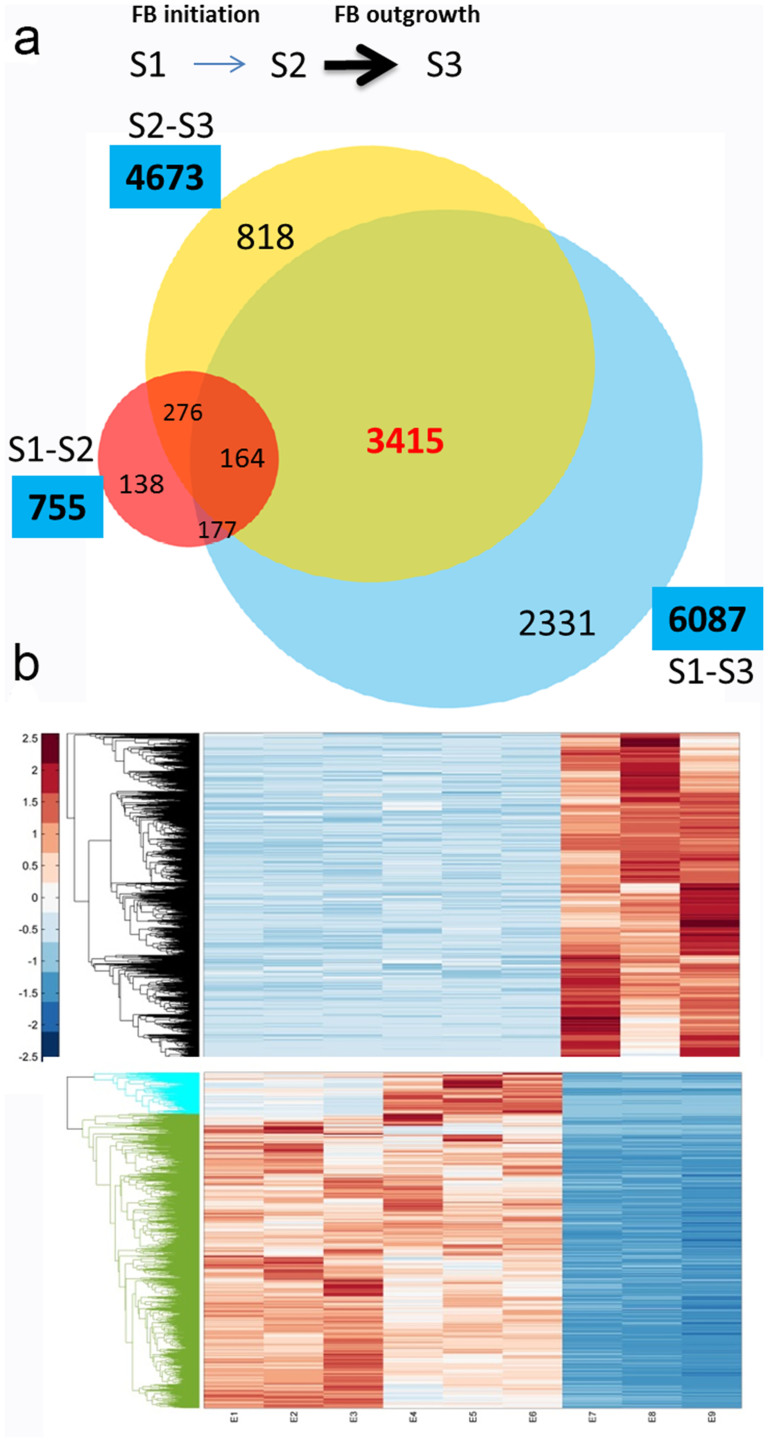
Analysis of DEGs between floral developmental stages. (a), A venn diagram of number of unique and common DEGs between different stages. (b), A hierarchical clustering graph of DEGs found between S1–S3 and S2–S3 but not S1–S2. Upper panel showing the DEGs with highest expression levels at stage 3; lower panel showing two clusters with lowest expression levels at stage 3.

To investigate molecular differences between developmental stages, we required to identify DEGs between samples. Expression levels were compared between stages to identify significant DEGs by applying cutoff of p-value < 0.05 (FDR Bonferroni corrected). A venn diagram of distribution of DEGs was shown (Fig. 5a), and we found that there were 755, 4637 and 6087 DEGs in S1–S2, S2–S3 and S1–S3 respectively (Fig. 5a). The numbers of DEGs in S2–S3 and S1–S3 were remarkably greater than S1–S2 indicating the involvement of complex developmental events at stage 3. A large proportion of DEGs between S1–S3 and S2–S3 were overlapped containing 3415 unigenes (Fig. 5a) suggesting it was specifically involved in the developmental processes at stage 3. To characterize this portion in detail, we further clustered this group of genes. And we found there are two major clusters of expression patterns-first one with apparent highest expression at stage 3, and second with lowest expression levels at stage 3 (Fig. 5b). The second cluster was also divided into two sub-groups with different expression between stage 1 and stage 2 (Fig. 5b). These results coincided well with markedly morphological changes during stage 3 and indicated various pathways of genes were involved in floral organ formation and bud outgrowth.

### GO enrichment analysis of DEGs captures differences of molecular events during floral bud development

**Figure 6:**
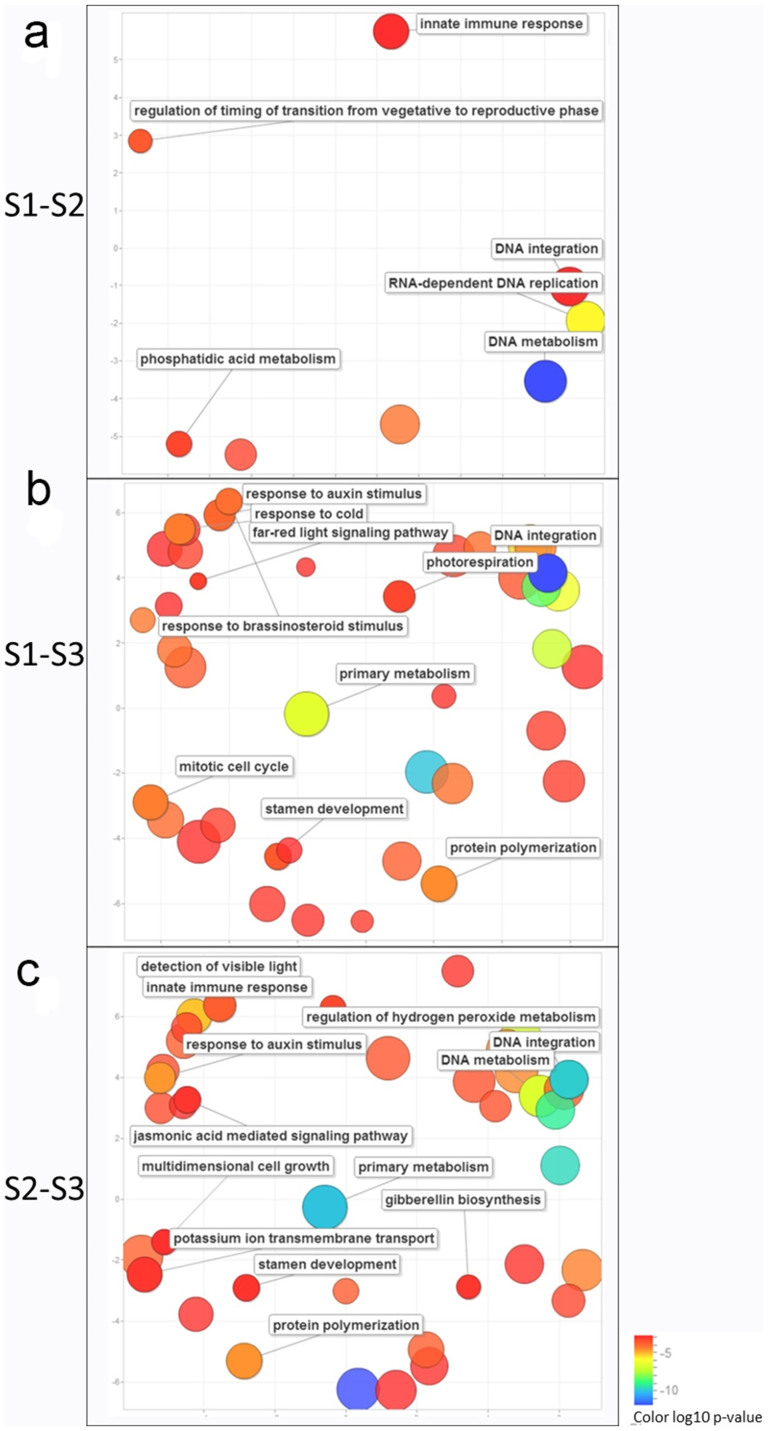
GO enrichment of DEGs between floral developmental stages. (a), the scatterplot of enriched GO terms between stage 1 and stage 2; (b), the scatterplot of enriched GO terms between stage 1 and stage 3; (c), the scatterplot of enriched GO terms between stage 2 and stage 3.

To depict the functional differences between developmental stages in *C. azalea*, the gene clusters of DEGs were characterized by GO enrichment analysis to explore the relevant biological functions. Based on our annotation of transcript, the GO terms of each DEG cluster were counted and evaluated by GO enrichment analysis^26^. The significant enriched GO terms (p-value < 0.05 FDR BH corrected) were identified (See for full list of GO terms in Supplementary table S3). Considering the hierarchy structures of GO systems, we used ReviGO tool to collectively visualize the enriched GO terms for each DEG cluster^27^. The comparisons of these GO term lists revealed that S1–S2 was different from S1–S3 and S2–S3 in terms of number and composition of GO terms (Fig. 6). And surprisingly, the GO term ('transition from vegetative to reproductive') was only identified in S1–S2 cluster (Fig. 6a). The GO terms of S2–S3 and S1–S3 were similar and both contained terms relevant to cold response, light signaling, and stamen development (Fig. 6b, c), but different in phytohormone signaling pathways. The brassinosteroid pathway was enriched in S1–S3, while auxin, jasmonic acid and gibberellin pathways were found in S2–S3 (Fig. 6b, c). These results were in good agreements with morphological observations as well as gene expression profiling, which suggested that specific regulators were required for the transition from vegetative to reproductive phase, and different developmental pathways were converged for orchestrating the development of floral bud.

### Differential expression patterns of key components in regulating flowering time

**Figure 7:**
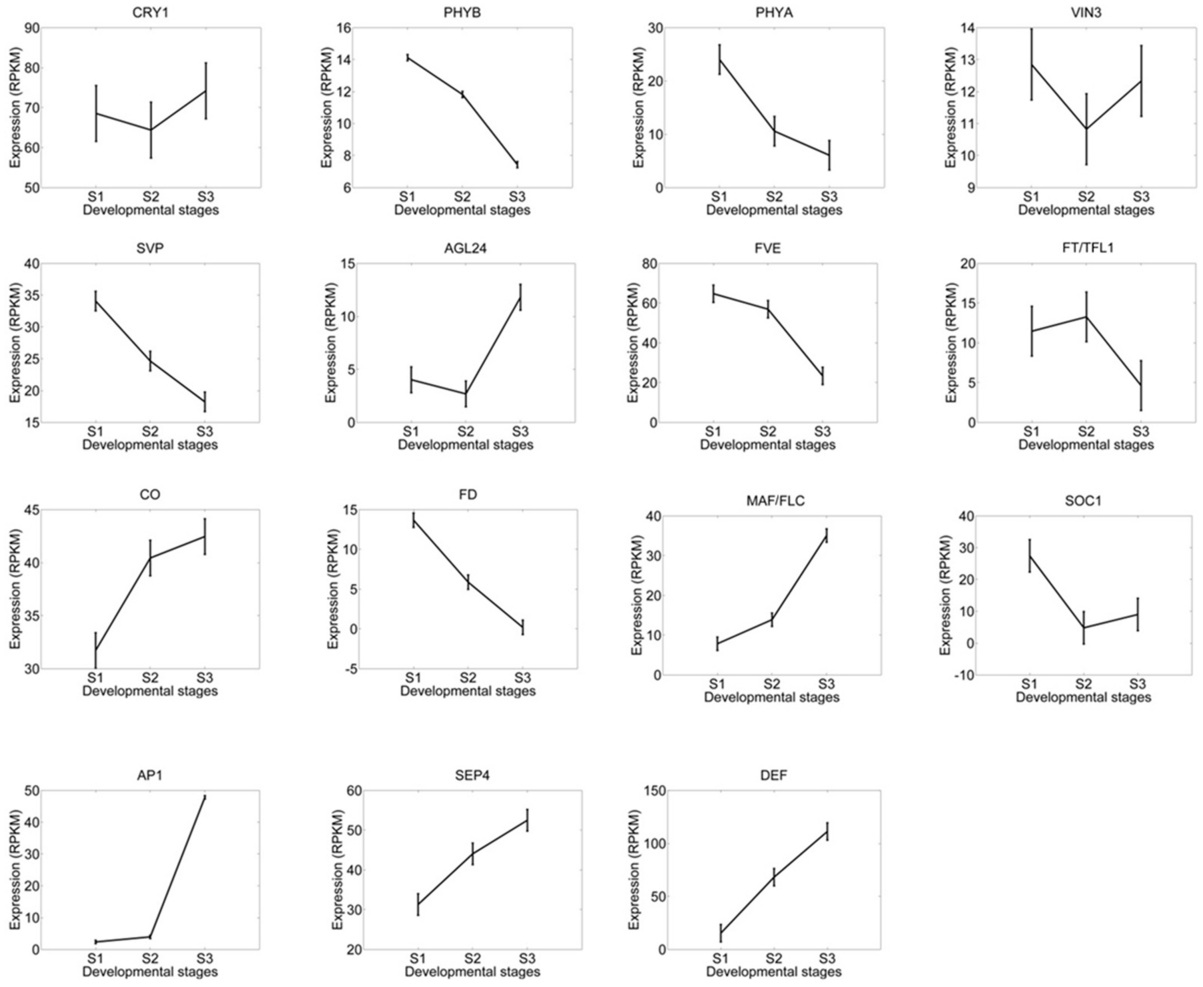
Expression patterns of key flowering regulators. Gene symbols were derived from TAIR and expression levels of putative orthologs were plotted. The mean values of RPKM of 15 orthologs of *cry1, phyB, phyA, VIN3, SVP, AGL24, FVE, FT/TFL1, CO, FD, MAF/FLC, SOC1, AP1, SEP4*, and *DEF* were plotted with standard deviations.

The GO enrichment analyses presented a top-down view of involvement of various biological processes during floral bud development. To explore how key floral regulators were related to the bud developmental process, we examined the expression patterns of key genes with relevance to flowering timing. We performed the reciprocal BLAST search analysis between^28^ coding gene database from *A. thaliana* and *C. azalea*, and a total of 25292 pairs of putative orthologs were identified (not shown). On the basis of orthologous analysis, genes related to flowering timing pathways were selected and characterized. We found that expression levels of genes related to red/far-red light signaling pathways (phyA, phyB) were decreasing during floral bud development suggesting light signaling was critical for initiation of floral bud at early stage (Fig. 7). Genes such as floral integrators and floral organ development (*CO*, *AP1*, *SEP4*, *DEF*) regulators displayed expected patterns that peaked in the late stage of bud development (stage 3) (Fig. 7), while with exceptions as *FD*, *SOC1* and *MAF*/*FLC* (Fig. 7). The blast search identified one transcripts (c75081.graph_c0) corresponding to both *FT* and *TFL1* which displayed decreasing pattern during floral bud development (Fig. 7). This indicated that this gene might function as a floral suppressor and thus is more similar to the Arabidopsis *TFL1*. These analyses revealed potential conserved and divergent function in the control of flowering time in different species.

### Gene family analysis of MADS-box genes identifies DAM genes in C. azalea

**Figure 8:**
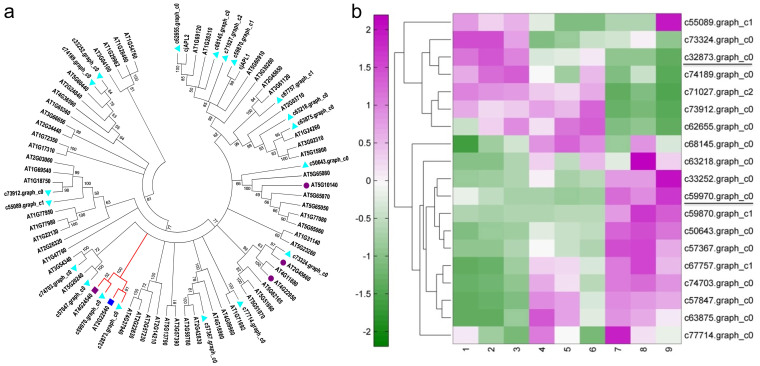
Phylogenetic and expression analyses of MADS-box genes. (a), A phylogenetic tree containing 19 MADS-box genes from *C. azalea* and 58 MADS-box genes from Arabidopsis as well as two MADS-box genes from *C. japonica*. Cyan triangles indicate genes from *C. azalea*. The red clade indicates the dormancy associated MADS-box genes. Red circles and color squares are corresponding to floral regulators in *Arabidopsis*: AT5G10140-*FLC/MAF*, AT2G45660-*SOC1*, AT4G22950-*AGL19*, AT4G24540-*AGL24*, AT2G22540-*SVP*. (b), Expression patterns of MADS-box genes from *C. azalea*.

The MADS-box transcription factors have been involved in many aspects of floral development and flowering timing^29^. To gather information of MADS-box gene family in *C. azalea*, we retrieved all members from *A. thaliana* and searched against database of *C. azalea* as mentioned above. In total we found 19 members of MADS-box gene family in *C. azalea* (Fig. 8). A phylogenetic tree containing MADS-box genes in *A. thaliana* and *C. azalea* and two AP1/FUL members from *Camellia japonica*^30^ was made (Fig. 8a), in which two orthologs of *SVP* and *AGL24* were identified forming a clade as DAM genes (Fig. 8a). The expression level of *SVP*- like gene decreased along bud development, while *AGL24*-like gene increased, indicating distinct roles of regulating floral development. The expression patterns of 19 MADS-box genes were clustered (Fig. 8b), and two major clusters were revealed displaying different patterns.

## Discussion

*C. azalea* is the only summer-flowering species in *Camellia* genus. Here we reported a comprehensive transcriptome study to characterize the gene expression profiles during bud development. The genomics resources are pivotal for comparative genomics analysis between economic *Camellia* species for the improvement of breeding programme, and also for understanding the bud development in perennial woody species. Through analysis of genome-wide expression patterns in three key developmental stages of flower, we identified differentially expressed genes and characterized the functional characteristics of DEG sets between different developmental transitions. The analyses captured the molecular signatures during floral bud development in *C. azalea*, which could be further exploited to help understand bud development in response to seasonal cues in trees.

### Genome-wide expression profiling uncovered developmental modules in response to seasonal cues

Due to the significance of plant reproduction, perennial plants have evolved a sophisticated system to detect environmental conditions and to regulate developmental programs^31^. Seasonal changes in day length and temperature are thought to be critical determinants of flowering in tree species^2^,^31^. In summer-flowering *C. azalea*, the floral buds can be found since spring till the end of year^32^. We examined the floral bud morphologies (Fig. 1), and found that the growth pattern of bud enlargement was related to floral developmental stages. Unlike *C. japonica* in which mature floral buds could be arrested for several months before flowering, *C. azalea* had a short period from bud formation to flowering (around 30 days), and recurrent bud formation and flowering could last over 9 months^32^. Based on histological analysis, we proposed that the three sampling stages were corresponding to floral bud initiation, floral organ differentiation, and bud outgrowth (Fig. 1). The expression patterns of floral integrators and organ development genes (e.g. CO, *AP1*, *SEP4*, *DEF*) peaking in the bud out growth stage supported the developmental process (Fig. 6). However, other orthologs such as *FD*, *SOC1* and *MAF/FLC* displayed differential patterns (Fig. 6) comparing to the expressions in *A. thaliana*^4^,^14^, suggesting potential functional diversifications between annuals and perennials. *FLC* was a major flowering suppressor in *A. thaliana*, and we identified one homolog (c50643_graph_c0) belonging to *MAF/FLC* clade displaying increased expression levels during bud development (Fig. 6). A study in perennial species *Arabis alpina* indicated that to achieve cyclical habitats, the repeated suppression and activation of *PERPETUAL FLOWERING 1* (*PEP1*), an ortholog of *FLC*, were required^33^. Whether *MAF*/*FLC* homologs in *C. azalea* are responsible for the unique flowering time and how it is related to environmental regulations of flowering require further investigations.

Comparisons of gene expression between developmental stages have revealed 755, 4673 and 6087 of DEGs between S1–S2, S2–S3, and S1–S3 respectively (Fig. 4). It indicated that between stage 1 and stage 2 there were much less DEGs than comparing to stage 3. This result was in agreement with markedly morphological alterations at stage 3 (Fig. 1). The outgrowth of floral bud involved various processes such as sugar and starch biosynthesis, cell wall expansion, cell cycle regulation and stress adaptation etc., to coordinate collectively for organogenesis^2^,^34^. The GO enrichment analyses of DEGs of S1–S3 and S2–S3 supported this notion that functional categories including light and stress signaling, DNA metabolism, cell cycle, floral organ development, and plant hormone pathways were significantly enriched (Fig. 5). However, between stage 1 and stage 2, most of these aforementioned GO categories were not identified, which was potentially related to subtle morphological changes (Fig. 1). Interestingly, functional category of 'regulation of timing of transition from vegetative to reproductive phase' was enriched only between stage 1 and stage 2 (Fig. 5a), suggesting conserved functions of flowering timing genes were required for the transition. Therefore, we demonstrated that we were able to detect molecular signatures by global analysis of gene expression and provide insights on the regulatory pathways in the control of floral bud development.

### Multiple pathways found altered in C. azalea buds could be associated to bud dormancy

In perennial woody species, it is critical to determine the specific timing of flowering according to seasonal changes. For instance, in *Populus*, a period of low temperatures and long days was required to break bud dormancy and initiate flowering^35^. *C. azalea* is the rare species discovered in genus *Camellia* that flowers during late spring to summer^23^. In this study, we showed that orthologs to *SVP* and *AGL24* displayed distinctive expression patterns during floral bud development (Fig. 6, 7) supporting their opposite roles in flowering. In peach, genes related to *SVP*/*AGL24* clade were characterized as DAM genes which were shown to be required for dormancy maintenance^12^. The DAM genes in perennials were thought to be regulated by seasonal cues^16^, so detailed expression analyses (i.e. seasonal expression) of DAM genes may provide more insights about the transcriptional regulation. It is not clear whether *SVP*- and *AGL24* like genes are involved in dormancy regulation. Characterizations of DEGs at stage 3 also identified phytohormones (auxin, jasmonic acid, gibberellin and brassinosteroid) pathways were involved in floral bud development (Fig. 6). All these phytohormones have been implicated in bud dormancy release in woody species by physiological and genetic analyses^36^,^37^. Hence, to understand the flowering timing in woody perennials the genetic framework from annuals need to be integrated with studies of bud dormancy. Systems biology approaches have greatly facilitated researches in non-model perennial species which gave important information of regulatory genes and pathways^38^,^39^. In this study we identified nine different clusters of co-expression genes (Fig. 5a), which can be further studied for comparative studies in other *Camellia* species to help improve breeding system. *C. japonica* is a popular ornamental flower species which has a long domestication history^30^, so the knowledge from *C. azalea* may lead to new cultivars with distinctive flowering periods.

### The special flowering timing of C. azalea

*C. azalea* has been mistakenly recognized as a species of azalea (*Rhododendron*) for many years due to its unique flowering period^22^. The adaptation of flowering pathway in *C. azalea* is still elusive. In this work we presented a detailed transcriptomics study to capture the dynamics of gene expression profiles during floral bud development. It would help us to better understand the regulation of flowering timing. However, to unlock the mechanism underpinning the switch of flowering habitat requires detailed comparative analyses from different closely related *Camellia* species, such as *C. japonica*. Future studies of comparisons of gene expression profiles at different bud development states across several *Camellia* species may shed some lights into the mechanism of flowering timing regulation. The morphologies of leaves and flowers of *C. azalea* were also slightly different from other *Camellia* species (i.e. *C. japonica*), which suggested other pathways of plant development might be involved as well. Overall, studies in *C. azalea* and other *Camellia* species have demonstrated an excellent system to investigate mechanism of plant adaptations to seasonal cues.

## Methods

### Plant material and RNA extraction

Camellia materials used in this study were grown in the greenhouse of Research Institute of Subtropical Forestry located in Fuyang (119°57′N, 30°04′ E; Fuyang city, Zhejiang, China) under natural light condition. For collecting samples of RNA, healthy floral buds or organs at different developmental stages were collected and frozen immediately in liquid nitrogen and stored in -80°C freezers before use. Three biological replicates were collected from three individuals, and each biological replicate contained 5–10 floral buds. For collecting floral buds at different developmental stages, the seasonal flowering habit of *C. azalea* was characterized as described^32^. The floral buds at different sizes were measured and histologically analyzed, and three stages of floral bud development: floral bud initiation, floral organ differentiation, and bud outgrowth, were identified for sampling. The exact time of collecting samples of the three stages was April 8^th^, April 22^nd^, June 13^th^ in the year of 2013. Total RNA was extracted from floral buds by using the Column Plant RNAout2.0 kit and treated with Column DNA Erasol (Beijing Tiandz Gene Technology Company, Beijing, China) to avoid the DNA contamination. RNA quality and quantity was determined using Nanodrop 1000 spectrophotometer (Thermo Fisher Scientific, Wilmington, DE) and Bioanalyzer RNA nano chip (Agilent Technologies, Singapore). Only the RNA samples with 260/280 ratio between 1.8 to 2.0, 260/230 ratio between 2.0 to 2.5 and RIN (RNA integrity number) more than 8.0, were used for sequencing.

### Illumina sequencing and de novo assembly

Approximately, 5 μg of total RNA for each tissue sample was used for the construction of libraries using mRNA-Seq Sample Prep kit (Illumina Inc., San Diego, CA) according to the manufacturer's protocol. Equal quantities of libraries (approximately 5 ng per sample) with different indices were mixed and stored in −80°C freezer before sequencing. Sequencing was performed in a v3 flowcell on an Illumina HiSeq 2000 sequencer, using the TruSeq Paired-End Cluster Kit v3 (Illumina PE-401-3001) and the TruSeq SBS HS Kit v3 200 cycles (Illumina FC-401-3001), generating 2 × 101 bp and 1 × 60 bp reads. Image analysis and base calling was done using the HiSeq Control Software version 1.4 and the Off-Line Base Caller v1.9. ~ 120 million high quality RNA-Seq reads (with quality score > 20 for each base) were pooled from Illumina sequencing of each of the 9 samples (three biological replicates of 3 stages) and were then assembled into contigs using Trinity (Release2012-04-27)^40^. All sequencing data were deposited in NCBI Short Read Archive under BioProject ID PRJNA257896 (accession number SRP045386). We quantified transcript levels in reads per kilobase of exon mode per million mapped reads (RPKM)^25^.

### Sequence annotation

The assembled sequences were compared against the National Center for Biotechnology Information (NCBI) non-redundant protein (Nr) database, NCBI non-redundant nucleotide sequence (Nt) database, and Swiss-Prot database using BlASTn (version 2.2.14) with an E-value cutoff at 10^−5^. Searches were limited to the first 10 significant hits for each query to increase computational speed. Open reading frames (ORFs) were predicted using the “getorf” program of EMBOSS software package^41^, with the longest ORF extracted for each unigene.

To identify gene ontology (GO) terms describing biological processes, molecular functions, and cellular components, the Swiss-Prot BLAST results were imported into Blast2GO^42^. These GO terms were assigned to query sequences, and produced a broad overview of groups of genes catalogued in the transcriptome for each of the three ontology vocabularies, namely, biological processes, molecular functions, and cellular components. The unigene sequences were also aligned to the Clusters of Orthologous Group (COG) database to predict and classify functions^43^.

### Differential gene expression and GO enrichment

The differential expression analysis was carried out by using the Bioconductor package EdgeR^44^. The right-sided hypergeometric enrichment test was performed at a medium network specificity selection and p-value correction was performed using the Benjamini-Hochberg method. The GO terms with p-values less than or equal to 0.05 were considered significantly enriched. The biological processes of enriched GO terms were visualized by using ReviGO^45^. And relatives of enriched GO terms were identified and plotted by using Matlab Bioinformatics toolbox (Mathworks Inc.).

### Clustering analysis

K-Means clustering was performed by Euclidean distance method and each centroid was the mean of the points in that cluster. Hierarchical clustering of gene expression was performed by clustergram function in Matlab Bioinformatics toolbox with default settings.

### Histological analysis

For histological sectioning, floral buds were fixed in formaldehyde-acetic acid (50% [v/v] ethanol, 3.7% [v/v] formaldehyde, 5% [v/v] acetic acid), dehydrated in an ethanol series, and embedded in paraplast (Sigma). Sections of 7-μm thickness were stained with safranin. All microscopy examinations were done with an Olympus BX-51 microscope (Olympus Corporation, Japan).

### Phylogenetic analysis

The MADS-box genes from Arabidopsis and *C. japonica* were derived as describes before^30^. Phylogenetic trees were made by MEGA5 using NJ method according to the manual^46^.

## Author Contributions

Z.F., J.L., Z.L. and H.Y. conceived and designed the study. Z.F. performed most of the experiments. Z.F., X.L., B.W. and J.W. conducted the sampling and histological analysis. Z.F. and H.Y. processed and analyzed the data. Z.F., H.Y., Z.L. and J.L. wrote the manuscript.

## Supplementary Material

Supplementary Information supplementary figures

Supplementary Information supplementary table1

Supplementary Information supplementary table2

Supplementary Information supplementary table3

## Figures and Tables

**Table 1:** Statistics of annotation analysis of unigenes

Annotation Database	Total Number	300 < = length < 1000	length > = 1000
COG	8855	2623	5471
GO	24086	9373	10698
KEGG	6128	2194	3051
Swissprot	21308	8433	9719
nr	32826	13725	13293
All	32999	13820	13303
