# Supplementary material for: Genome-wide transcriptome profiling provides insights into floral bud development of summer-flowering Camellia azalea
Source: Sci Rep. 2015 May 15;5:9729. doi: 10.1038/srep09729 (PMC4432871; doi:10.1038/srep09729)
Supplement: Supplementary Information — supplementary figures [file srep09729-s1.pdf]

Genome-wide transcriptome profiling provides insights into floral bud development of summer-flowering *Camellia azalea*

Zhengqi Fan[1,2], Jiyuan Li[1,2*], Xinlei Li[1,2], Bin Wu[1], Jiangyin Wang[1], Zhongchi Liu[3], Hengfu Yin[1,2*]

Authors' addresses:

1. Research Institute of Subtropical Forestry, Chinese Academy of Forestry, Fuyang, 311400, Zhejiang, China

2. Key Laboratory of Forest genetics and breeding, Zhejiang Province, China

3. Department of Cell Biology and Molecular Genetics, University of Maryland, College Park, Maryland, USA

*Corresponding authors: E-mail: ***hfyin@sibs.ac.cn*** or ***jiyuan_li@126.com***

Tel/Fax: +86-571-63346372

Supplementary Information

This file contains 2 supplementary figures and captions of three supplementary files.

Figure legends

Fig.S1. Analysis of functional categories of annotated transcripts. **A,** Distribution of number of transcripts against KEGG's A to Z function classes. **B**, Distribution of number of annotated transcripts in GO database.

Fig. S2. Distribution of correlation co-efficiencies between each pair of samples.

Figure S1

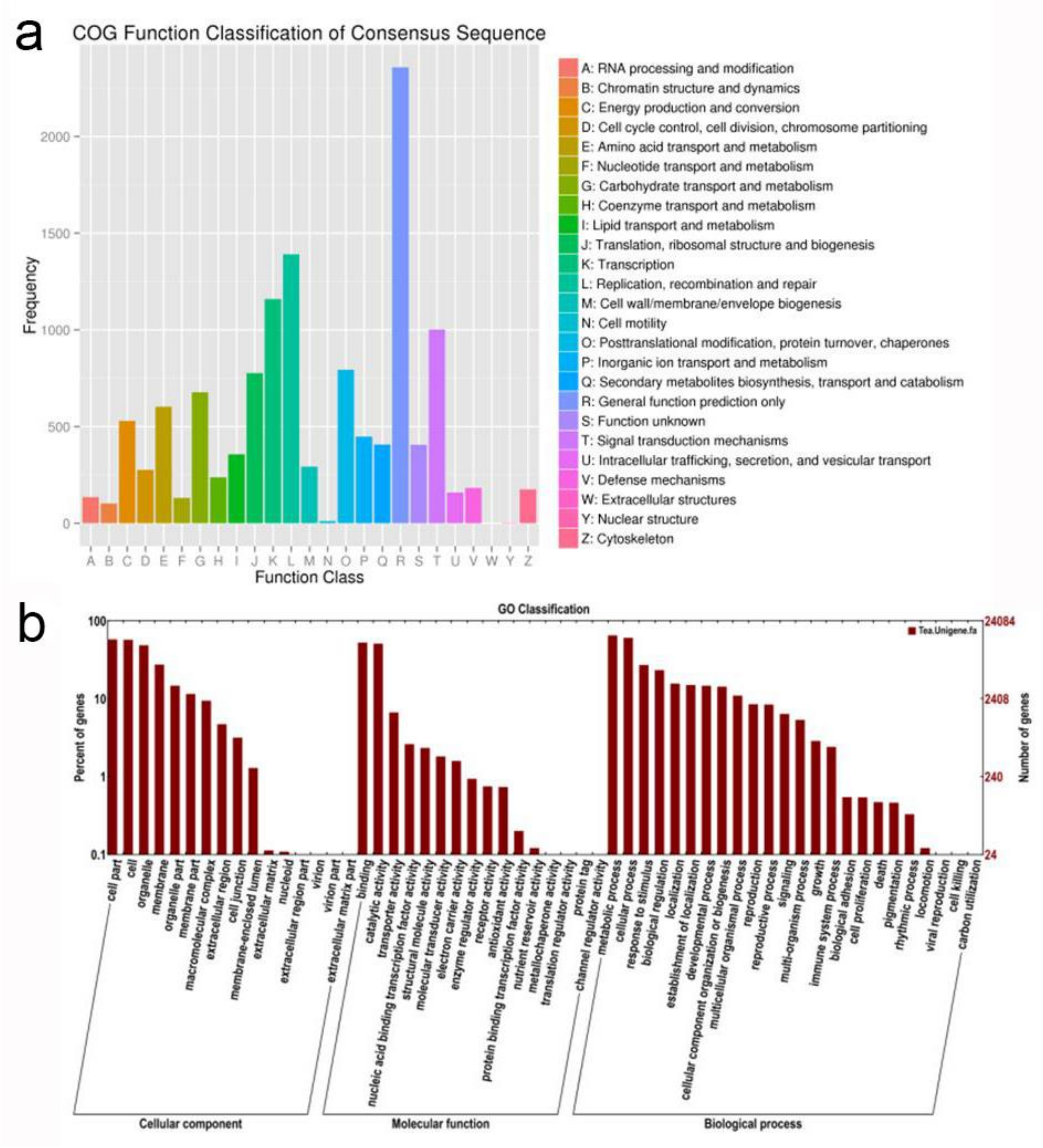

a
COG Function Classification of Consensus Sequence
Frequency
0
500
1000
1500
2000
Function Class
A B C D E F G H I J K L M N O P Q R S T U V W Y Z
A: RNA processing and modification
B: Chromatin structure and dynamics
C: Energy production and conversion
D: Cell cycle control, cell division, chromosome partitioning
E: Amino acid transport and metabolism
F: Nucleotide transport and metabolism
G: Carbohydrate transport and metabolism
H: Coenzyme transport and metabolism
I: Lipid transport and metabolism
J: Translation, ribosomal structure and biogenesis
K: Transcription
L: Replication, recombination and repair
M: Cell wall/membrane/envelope biogenesis
N: Cell motility
O: Posttranslational modification, protein turnover, chaperones
P: Inorganic ion transport and metabolism
Q: Secondary metabolites biosynthesis, transport and catabolism
R: General function prediction only
S: Function unknown
T: Signal transduction mechanisms
U: Intracellular trafficking, secretion, and vesicular transport
V: Defense mechanisms
W: Extracellular structures
Y: Nuclear structure
Z: Cytoskeleton
b
GO Classification
Tea.Unigene.fa
Percent of genes
100
10
1
0.1
Number of genes
24084
2408
240
24
cell part
cell
organelle
membrane
organelle part
membrane part
macromolecular complex
extracellular region
cell junction
membrane-enclosed lumen
extracellular matrix
nucleoid
extracellular region part
virion
virion part
extracellular matrix part
binding
catalytic activity
transporter activity
nucleic acid binding transcription factor activity
structural molecule activity
molecular transducer activity
electron carrier activity
enzyme regulator activity
receptor activity
antioxidant activity
protein binding transcription factor activity
nutrient reservoir activity
metallochaperone activity
translation regulator activity
protein tag
channel regulator activity
metabolic process
cellular process
response to stimulus
biological regulation
localization
establishment of localization
developmental process
cellular component organization or biogenesis
multicellular organismal process
reproduction
reproductive process
signaling
multi-organism process
growth
immune system process
biological adhesion
cell proliferation
death
pigmentation
rhythmic process
locomotion
viral reproduction
cell killing
carbon utilization
Cellular component
Molecular function
Biological process

Figure S2

	1	2	3	4	5	6	7	8	9
1	1.000								
2	0.988	1.000							
3	0.981	0.965	1.000						
4	0.953	0.934	0.946	1.000					
5	0.962	0.935	0.955	0.975	1.000				
6	0.978	0.971	0.972	0.966	0.964	1.000			
7	0.618	0.550	0.624	0.647	0.715	0.617	1.000		
8	0.602	0.534	0.608	0.636	0.700	0.602	0.984	1.000	
9	0.524	0.461	0.528	0.554	0.615	0.522	0.949	0.952	1.000

Supplementary Table S1 Annotation of unigenes of *C. changii.*

Supplementary Table S2 Quantification of transcripts in three developmental stages. Length of assembled transcripts was listed. E1-E3, three replicates of samples at stage 1; E4-E6, three replicates of samples at stage 2; E7-E9, three replicates of samples at stage 3.

Supplementary Table S3 Enriched GO terms of DEGs between developmental stages. P values and FDR corrected P values were listed.

Files can be downloaded at the online links.
